# Venenum bufonis and its active constituents alleviate RSV-induced pneumonia in mice by suppressing macrophage infiltration and NLRP3 inflammasome activation

**DOI:** 10.1016/j.virusres.2025.199672

**Published:** 2025-12-03

**Authors:** Hui Cai, Lijing Hou, Jingjie Chang, Qing Yang, Mingming Wang, Lin Wang, Yao Li, Xueting Cai, Jie Yang, Peng Cao, Jiao Chen

**Affiliations:** a Jiangsu Provincial Integrated Innovation Center of Hospital Preparations, Affiliated Hospital of Integrated Traditional Chinese and Western Medicine, Nanjing University of Chinese Medicine, Nanjing, Jiangsu, 210028, China; b State Key Laboratory on Technologies for Chinese Medicine Pharmaceutical Process Control and Intelligent Manufacture, Nanjing University of Chinese Medicine, Nanjing, Jiangsu, 210023, China; c Shandong Academy of Chinese Medicine, Jinan, 250014, China

**Keywords:** Venenum bufonis, Respiratory syncytial virus, Pneumonia, Macrophages, NLRP3

## Abstract

Respiratory syncytial virus (RSV) is a leading cause of lower respiratory tract infections in infants and the elderly, with limited treatment options. Venenum bufonis (Vb), a traditional Chinese medicine, exhibits broad pharmacological activities including antiviral and anti-inflammatory effects, but its potential against RSV-induced pneumonia remains unclear. This study evaluates Vb’s therapeutic effects and mechanisms in RSV-infected pneumonia and identifies its key active constituents. RSV pneumonia was induced in C57BL/6 mice via intranasal inoculation. Mice were treated with Vb or ribavirin intraperitoneally for five days. Lung pathology, viral gene, inflammatory cytokine gene expression, NLRP3 pathway activation, and immune cell infiltration were assessed using H&E staining, electron microscopy, RT-qPCR, immunohistochemistry, RNA sequencing and flow cytometry. The results demonstrated that Vb treatment significantly reduced lung tissue damage, mRNA levels of RSV-N protein, proinflammatory cytokines (TNF-α, IL-6) and type II interferon (IFN-γ), and enhanced the mRNA levels of type I interferons (IFN-α/β). Furthermore, Vb markedly decreased macrophage infiltration and suppressed mRNA expression of NLRP3, Caspase-1, and IL-1β in lung tissue, suggesting it may alleviate RSV pneumonia by inhibiting macrophage-driven inflammation and NLRP3 activation. Additionally, bufalin, cinobufagin, and resibufogenin were identified as likely bioactive constituents mediating Vb’s therapeutic effects. This study provides a scientific basis for the potential application of Vb in the treatment of RSV-induced pneumonia.

## Highlights

• Venenum bufonis significantly alleviates RSV-induced pneumonia in C57BL/6 mice, effectively suppresses the gene expression of RSV and pro-inflammatory cytokines in the lungs.
• Venenum bufonis alleviates RSV-induced pneumonia potentially via suppression of macrophage infiltration and NLRP3 inflammasome activation in the lungs.
• Bufalin, cinobufagin and resibufogenin are the key active constituents in Venenum bufonis that attenuate RSV-induced pneumonia.
• This study identifies Venenum bufonis as a potential therapeutic medicine for RSV-associated viral pneumonia, providing a possible strategy for clinical intervention.

## 1 Introduction

Respiratory syncytial virus (RSV) is an enveloped, negative-sense, single-stranded RNA virus of the Orthopneumovirus genus of the Pneumoviridae family in the order Mononegavirales (Duan et al., 2024; Groves et al., 2018). Its pathogenic mechanism mainly includes the destruction of host cell structure, viral entry and replication. RSV exhibits high transmissibility primarily via close contact and respiratory droplets, rendering its effective prevention challenging in clinical and community settings (Shi et al., 2016). RSV infection triggers profound respiratory pathology characterized by mucus hypersecretion and robust inflammatory cascades, ultimately resulting in airway obstruction and pulmonary dysfunction. As a leading global pathogen, RSV disproportionately affects vulnerable populations including infants, immunocompromised individuals, and older adults, causing severe lower respiratory tract infections (Meissner et al., 2004). The vast majority of children are infected with RSV before the age of two. Given its high prevalence and substantial disease burden, RSV poses a major public health challenge. Furthermore, the resurgence of RSV transmission in China, Europe, and the United States has imposed a significant societal burden (Shao et al., 2022). Recurrent RSV infections are common, primarily attributable to the virus’s ability to evade immune responses through multifaceted escape mechanisms (Christiaansen et al., 2015; Larrañaga et al., 2009). These repeated infections jeopardize respiratory health and may adversely affect long-term health in affected individuals (Haddadin et al., 2021).

Current clinical management of RSV infection is primarily based on symptomatic treatment, typically involving bronchodilators, epinephrine, corticosteroids, and hypertonic saline solutions (DeVincenzo et al., 2015, 2014; Rodriguez and Ramilo, 2014). Ribavirin remains the only approved antiviral agent for RSV infection. However, due to its adverse effect profile, ribavirin administration is typically restricted to adjunctive therapy in severe cases (Gatt et al., 2023; Langedijk and Bont, 2023). Consequently, there is an urgent need to develop novel therapeutics capable of mitigating RSV-induced viral pneumonia.

As a classical traditional Chinese medicine material, Venenum bufonis (Vb) is primarily sourced through the desiccation of glandular secretions from two Bufonidae species, *Bufo gargarizans Cantor* and *Bufo melanostictus Schneider*, which demonstrates remarkable pharmacological activities (Gowda et al., 2003). Within traditional medical systems, Vb have been historically employed for their cardiovascular effects, notably as cardiac stimulants and diuretics (Jia et al., 2022). Vb is documented to possess multiple therapeutic properties, including detoxification, anti-edema, analgesia, spleen-strengthening, and resuscitative effects. Based on this, various formulations containing Vb as the main ingredient, such as Liushen Pill, Chansu Injection, and Shexiang Baoxin Pill, have been clinically applied to treat a variety of diseases, including cutaneous abscesses, pharyngeal inflammations, syncope, and as adjuvant therapy for tumors (Xu et al., 2021). Vb has been employed for millennia across East Asian traditional medicine systems, particularly in China, Japan, and Southeast Asia, demonstrating significant therapeutic applications as a cardiotonic, antimicrobial, and analgesic agent. Historical records also document its use during epidemic outbreaks (Qi et al., 2013).

The primary bioactive constituents of Vb are bufadienolides, including bufalin, cinobufagin, and resibufogenin. Current studies demonstrate that bufalin, the principal bioactive constituent, blocks host cell entry of murine hepatitis virus (MHV), feline infectious peritonitis virus (FIPV), and Middle East respiratory syndrome coronavirus (MERS-CoV) by targeting the Src signaling pathway mediated by Na^+^/K^+^-ATPase α1 subunit (ATP1A1) (Burkard et al., 2015). Preliminary clinical observations from our research group indicate that Vb exhibited significant therapeutic efficacy in patients with severe COVID-19, notably improving oxygenation indices and respiratory function, and reduced the duration of respiratory support (Hu et al., 2021). No treatment-emergent adverse events were observed during the study period. These studies indicate that Vb possesses broad-spectrum antiviral activity, highlighting its potential as a therapeutic candidate (García-Dorival et al., 2014). However, the antiviral activity of Vb and its principal active constituents against RSV-associated pneumonia remains unclear. Our findings in this study established that Vb and its main constituents bufalin, cinobufagin and resibufogenin significantly ameliorated RSV-induced pulmonary tissue damage in mice while reducing viral gene and pro-inflammatory cytokines expression, and enhancing the expression of type I interferons (IFN-α/β) to combat viral infection. This protective effect may be attributed to the suppression of macrophage infiltration and NLRP3 inflammasome activation. Our study provides a scientific basis for the potential application of Vb in the treatment of RSV-induced viral pneumonia.

## 2 Materials and methods

### 2.1 Reagents

The Vb used in this study was a sterilized aqueous solution prepared by extracting the dried secretion of *bufo gargarizans Cantor* with 75 % ethanol (National Drug Approval No. Z32020693, generic name: Chansu injection), provided by Jiangsu Pujin Pharmaceutical Co., Ltd. Bufalin (T1719), cinobufagin (T5A2461) and resibufogenin (T4A2458) and CCK-8 (C0005) were purchased from TargetMol. Ribavirin (HY-B0434) were purchased from MCE. eBioscience™ Fixable Viability Dye eFluor™ 506 (65–0866–14) were purchased from Thermo Fisher Scientific. Purified anti-mouse CD16/32 (101302), anti-mouse PE-Cy7 CD45 antibody (103114), anti-mouse FITC CD3 antibody (100204), anti-mouse PE CD4 antibody (100407), anti-mouse APC CD8 antibody (100711), BD Horizon™ RB705 rat anti-mouse F4/80 (570288), BD Pharmingen™ APC rat anti-CD11b (561690) were purchased from Becton Dickinson. Fetal bovine serum (C04001–050) and sterile phosphate buffered saline (C3580–0500) were purchased from Viva Cell. Penicillin-streptomycin (15140122), RPMI-1640 Medium (C11875500BT) and Dulbecco's modified eagle medium (DMEM, C11995500BT) were purchased from Gibco. HiScript II Q RT SuperMix for qPCR with gDNA wiper (R223), VeZol reagent (R411–01), and ChamQ SYBR Color Qpcr Master Mix (Q431) were purchased from Nanjing Vazyme Biotechnology Co., Ltd. Type IV collagenase (LS004188) were purchased from Worthington. Lipopolysaccharide (LPS, L2603) were purchased from Sigma-Aldrich.

### 2.2 Cells and virus

The human Hep-2 cell line (CRL-9609, ATCC) were grown in RPMI-1640 medium supplemented with 1 % penicillin/streptomycin and 10 % heat-inactivated FBS, in a humidified 5 % CO₂ atmosphere at 37 °C. RSV isolate was obtained from the Institute of Basic Medical Sciences at Shandong Academy of Medical Sciences, China. The murine macrophage cell line RAW 264.7 (obtained from the Cell Resource Center, Institute of Basic Medical Sciences, Chinese Academy of Medical Sciences) was cultured in DMEM supplemented with 10 % fetal bovine serum (FBS) and 1 % penicillin-streptomycin. Cells were maintained at 37 °C in a humidified atmosphere containing 5 % CO₂.

To determine the effect of Vb on the viability of RAW 264.7 cells, a CCK-8 assay was performed. Following overnight seeding of cells in 96-well plates (5 × 10³ cells/well), the culture medium was replaced with fresh medium containing Vb at concentrations ranging from 0.3125 to 20 ng/mL. After a 24 h incubation, 10 µL of CCK-8 solution was added to each well, and the plates were incubated for another 1 h before the absorbance was measured at 450 nm. Cell viability was calculated relative to the negative control (drug-free medium) and the blank control (medium only).

To establish an in vitro inflammation model, the cells were stimulated with 100 ng/mL LPS for 24 h. Following stimulation, the LPS-containing supernatant was aspirated and replaced with fresh medium containing various concentrations of Vb (20, 15, 10, 5, 2.5, 1.25, 0.625 and 0.3125 ng/mL) for an additional 24 h co-incubation period. Cells in the blank control group and the LPS-only group were included as controls.

### 2.3 Animal modeling and drug administration

Male C57BL/6 mice (3–4 weeks old, specific pathogen-free (SPF) grade) were purchased from Beijing Vital River Laboratory Animal Technology Co., Ltd. All animals were housed in the laboratory of the Animal Experiment Center at Shandong University of Traditional Chinese Medicine under controlled conditions: constant temperature (22 ± 2 °C), humidity (50 ± 10 %), and a 12 h light/dark cycle, with ad libitum access to food and water. All animal experiments were approved by the Institutional Animal Care and Use Committee of Shandong University of Traditional Chinese Medicine (Approval No. SDUTCM20241109101) and were conducted in strict accordance with the Animal Research: Reporting of In Vivo Experiments (ARRIVE) guidelines. The mouse pneumonia model was established through intranasal inoculation with RSV. Mice in the RSV model group and all therapeutic intervention groups were inoculated intranasally with 100 TCID_50_ of viral suspension, while the vehicle group received an equal volume of 2 % DMEM via identical inoculation procedures. Pharmacological intervention was initiated 24 h after model establishment.

In our present study, the dose of 12 μg/kg used in mice corresponds to the clinically equivalent therapeutic dose. The dose of 24 μg/kg is twice the clinically equivalent therapeutic dose. The dosage and administration method of the positive drug ribavirin 50 mg/kg were based on the references we consulted (Shi et al., 2016). The animals were divided into Vb groups which received Vb at doses of 12 μg/kg, 24 μg/kg, and Ribavirin group which was treated with 50 mg/kg ribavirin as positive control. All treatments were delivered in a consistent volume of 100 μL via intraperitoneal injection. The vehicle group and RSV model group received intraperitoneal injections of PBS in volumes equivalent to the administered treatments. All groups received once-daily injections for five consecutive days. On day 5 post-treatment, mice were humanely euthanized by cervical dislocation followed by immediate lung tissue collection. The right lung was allocated for comprehensive pathological evaluation including histopathological examination, immunohistochemical analysis, viral load quantification, and inflammatory cytokines detection, while the left lung was preserved for ultrastructural analysis through electron microscopy and subsequent immune cell infiltration assessment. In the active constituent ingredient identification experiment, two dose groups were set for bufalin, cinobufagin and resibufogenin (0.1 mg/kg and 0.5 mg/kg, respectively), with all other experimental procedures performed identically to the Vb group.

### 2.4 Histopathological evaluation and immunohistochemistry

A portion of the right lung tissue was fixed in 4 % paraformaldehyde and processed for paraffin embedding. The tissue sections underwent dewaxing and hydration before being treated with high-definition constant staining pretreatment solution for 1 min. Subsequent staining procedures included hematoxylin staining followed by eosin staining. Following dehydration and sealing, microscopic examination was performed with image acquisition and analysis to evaluate the pathological changes in the lung tissues. Additional paraffin-embedded sections from the same anatomical region of lung tissues were processed through standard deparaffinization and rehydration procedures. Then sequentially perform antigen repair, endogenous peroxidase blocking, serum blocking, primary and secondary antibodies incubation,3,3′-diaminobenzidine (DAB) color development, nuclei restaining, dehydration and sealing. Finally, observe the results under a bright-field microscope to analyze the expression levels of inflammatory cytokines in the lung tissues of mice from each group.

### 2.5 Transmission electron microscopy analysis

Approximately 1 mm³ fragment of fresh left lung tissue was dissected and immediately placed into 1.5 mL EP tubes containing electron microscopy fixative for primary fixation, followed by post-fixation processing, dehydration at room temperature, resin penetration and embedding, polymerization, positioning, ultrathin sectioning and staining. Finally, the samples were observed under transmission electron microscopy (TEM), with subsequent image acquisition and analysis.

### 2.6 Real-time PCR analysis

RNA extraction was carried out using VeZol Reagent and cDNAs were synthesized with a HiScript II Q RT SuperMix for qPCR with gDNA Wiper. Real-time PCR analysis was performed using ChamQ SYBR Color Qpcr Master Mix. Primer sequences (Forward and Revers 5′−3′) are detailed in Table 1.

**Table 1:** The primer sequences used in real-time PCR analysis.

Gene	Forward (5′−3′)	Reverse (5′−3′)
Mouse *GAPDH*	CCACAGCCTTGGCAGCACCA	ATCTCCGCCCCTTCTGCCGA
Mouse *β-actin*	CGTTGACATCCGTAAAGACC	AACAGTCCGCCTAGAAGCAC
Mouse *IL-6*	TGCAAGTGCATCATCGTTGTTC	CCACTTCACAAGTCGGAGGC
Mouse *IL-1β*	ATCTTTTGGGGTCCGTCAACT	GCAACTGTTCCTGAACTCAACT
Mouse *TNF-α*	CCATGCCGTTGGCCAGGAGG	ATCCGCGACGTGGAACTGGC
Human *RSV-N*	GCAGTGCAGTTAGCAAAGGC	GCGATTGCAGATCCAACACC
Mouse *NLRP3*	ATTACCCGCCCGAGAAAGG	CATGAGTGTGGCTAGATCCAAG
Mouse *Caspase-1*	ACAAGGCACGGGACCTATG	TCCCAGTCAGTCCTGGAAATG
Mouse *IFN-γ*	GGATGCATTCATGAGTATTGC	CCTTTTCCGCTTCCTGAGG
Mouse *IFN-α*	AAGTATTTCCTCACAGCCAGCAG	AGTCCATCAGCAGCTCAATGAC
Mouse *IFN-β*	CAGCTCCAAGAAAGGACGAAC	GGCAGTGTAACTCTTCTGCAT

### 2.7 Flow cytometry analysis for macrophages and T lymphocytes infiltration

Single-cell suspensions were prepared by enzymatic digestion of minced lung tissues with type IV collagenase (1 mg/mL) in RPMI 1640 medium at 37 °C for 30 min with 200 rpm agitation. The reaction was terminated by adding PBS buffer containing 2 % FBS. Digested lung tissues were mechanically disrupted by passage through a 70 μm sterile strainer. The filtered sample was treated with red blood cell lysis buffer for 2 mins, followed by terminating the reaction with PBS buffer supplemented with 2 % FBS. After washing with ice cold PBS, the cells were collected and resuspended in PBS, and then stained with PE-cy7-labeled anti-CD45, FITC-labeled anti-CD3, PE-labeled anti-CD4, APC-labeled anti-CD8, RB705-labeled anti-F4/80 and APC-labeled anti-CD11b. The samples were analyzed on BD AccuriTM C6 PlusFlow Cytometer. FlowJo7.6.1 software was used for data analysis.

### 2.8 In vitro antiviral assay against RSV

We evaluated the anti-RSV activity of Vb through an in vitro cell protection assay monitoring virus-induced cytopathic effect (CPE). Hep-2 cells grown to monolayers in 96-well plates were infected with RSV at an MOI of 0.25 (100 μL virus inoculum in RPMI-1640 medium) while simultaneously treating with 100 μL of drug-containing maintenance medium. Following infection at 37 °C, characteristic CPE progression including cell swelling, rounding, reduced refractivity and syncytium formation were observed. After 5 days incubation when virus controls showed 75–100 % CPE, cell viability was quantified using the CellTiter-Glo® Luminescent assay. The inhibition rate was calculated as: [(Drug RLU - Virus control RLU)/ (Cell control RLU - Virus control RLU)] × 100 %.

### 2.9 Determination of major constituents in Vb by HPLC

The major constituents of Vbused in this study was identified by high-performance liquid chromatography (HPLC). The analysis was performed using a Waters 2696 HPLC system equipped with a Phenomenex Luna 5μ C18(2) column (250 mm × 4.6 mm). The mobile phase consisted of acetonitrile and 0.1 % ammonium acetate solution (50:50, v/v) with a detection wavelength set at 296 nm. The system suitability was verified by achieving a theoretical plate count of not less than 3000 calculated for the bufalin peak. This validated HPLC method was employed for the qualitative determination of the chemical constituents in Vb.

### 2.10 RNA-seq and data analysis

Total RNA was extracted from the lung tissues of RSV-infected model and Vb (24 μg/kg) treatment mice (*n* = 3 per group) using VeZol reagent. RNA quality was verified by agarose gel electrophoresis, a NanoPhotometer spectrophotometer, and an Agilent 2100 bioanalyzer. Sequencing libraries were prepared with Illumina's NEBNext® Ultra™ RNA Library Prep Kit, quantified via Qubit 2.0 Fluorometer, and assessed for insert size using the Agilent 2100. Qualified libraries were sequenced on an Illumina platform.

Differential expression analysis was performed using the DESeq2 R package (v1.16.1), with genes under an adjusted p-value (FDR) < 0.05 considered statistically significant. The results were validated using the edgeR package (v3.18.1). Functional enrichment analysis of KEGG pathways for the identified differentially expressed genes (DEGs) was conducted using the clusterProfiler R package (v3.4.4).

### 2.11 Data statistical analysis

Unless otherwise stated, all the results are given as the means ± SEM of three separate studies. *P* value of <0.05 was considered significant. One-way analysis of variance (ANOVA) followed by a Tukey test was performed to compare more than two groups. All statistical analyses were performed using GraphPad Prism software (version 9.5.1).

## 3 Results

### 3.1 Vb alleviates RSV-induced pneumonia in mice by reducing lung injury and viral infection

The therapeutic effects of Vb on RSV infection-induced pneumonia were evaluated using a murine model. Twenty-four hours post-infection, mice received daily intraperitoneal injections of Vb or ribavirin for five consecutive days, then the lung tissues were collected for both histopathological analysis and viral mRNA quantification. Additionally, body weight of mice in all groups was monitored daily for 7 days throughout the modeling and treatment phases. No significant change in body weight was observed in the high-dose (24 μg/kg) Vb group compared to the RSV model group and the positive control group (ribavirin), suggesting the absence of apparent toxicity for Vb (Supplementary Fig. S1 A).

**Fig. 1:**
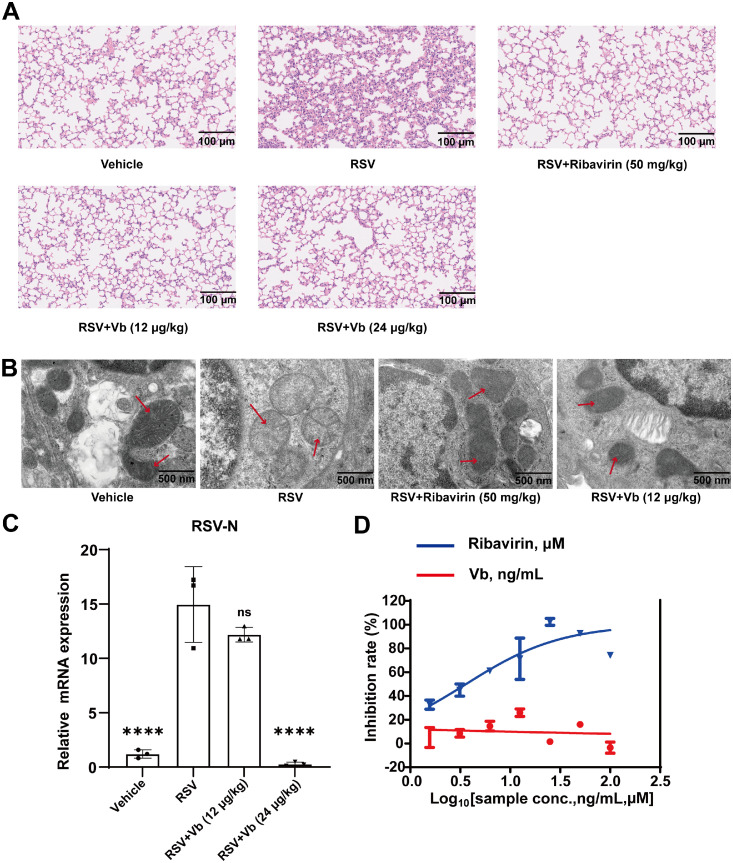
Vb alleviates RSV-induced pneumonia in mice. (A) Representative H&E-stained lung sections. Scale bar: 100 μm. (B) Representative TEM images showing ultrastructural features of lung tissues. Scale bar: 500 nm. The red arrows point to the mitochondria in the cytoplasm of type II pneumocytes. (C) Quantification of mRNA levels of RSV-N protein in murine lung tissues (*n* = 3). *P* < 0.05 indicates statistical significance versus the RSV model group, “ns” denotes no significant difference, ^⁎⁎⁎⁎^*P* < 0.0001. (D) In vitro evaluation of anti-RSV activity of Vb and ribavirin in Hep-2 cells, ng/mL for Vb and μM for ribavirin*.*

H&E staining revealed disrupted alveolar architecture in the model group, characterized by thickened alveolar septa, extensive inflammatory cell infiltration, alveolar congestion and hemorrhage, indicating severe pulmonary injury. In contrast, Vb treatment significantly attenuated these pathological changes. Compared with the low-dose (12 μg/kg) group, the high-dose (24 μg/kg) group of Vb demonstrated more pronounced therapeutic efficacy (Fig. 1A).

TEM analysis further demonstrated substantial ultrastructural damage to alveolar epithelial cell mitochondria in RSV-infected mice, inducing spherical mitochondrial morphology, pronounced matrix swelling, decreased electron density, and vacuolar degeneration. These findings indicate that RSV infection induces significant mitochondrial damage in alveolar epithelial cells. In contrast, the mitochondrial morphology in the Vb-treated group showed significant alterations compared to the model group, appearing rod-shaped or spherical with no obvious matrix swelling or vacuolar degeneration (Fig. 1B). These results demonstrate that Vb treatment effectively alleviates RSV infection-induced lung injury and mitochondrial damage in alveolar epithelial cells.

The nucleocapsid protein of the respiratory syncytial virus (RSV-N), is a core structural component essential for viral replication that encapsulates the viral RNA genome to form the nucleocapsid. In this study, we measured RSV-N mRNA expression in mouse lung tissue to evaluate the inhibitory effect of Vb on viral replication (Bakker et al., 2013; Liang et al., 2021; Richard et al., 2018). Vb (24 μg/kg) treatment significantly reduced RSV-N protein transcript level in murine lung tissues, indicating that Vb has the ability to decrease viral levels in the lungs of RSV-infected mice (Fig. 1C).

Furthermore, ribavirin exhibited dose-dependent inhibition of RSV replication in Hep-2 cells, while Vb displayed no significant antiviral activity at the tested concentrations (Fig. 1D). These findings suggest that the therapeutic effects of Vb may be mediated through indirect mechanisms, such as immunomodulation, rather than direct antiviral action.

### 3.2 Vb suppresses pro-inflammatory cytokine expression and macrophage infiltration in RSV-infected lungs

Interferons have been established as critical mediators of broad-spectrum antiviral defense and immune regulation. Type I interferon, primarily IFN-α and IFN-β, serve as the first line of defense against viral infections by inducing the production of various antiviral proteins in host cells (Darnell et al., 1994; MacMicking, 2012; McNab et al., 2015; Xu et al., 2025; Yan and Chen, 2012). Type II interferon IFN-γ functions as a potent enhancer and immunomodulator during antiviral defense. However, excessive or sustained IFN signaling can contribute to inflammatory pathology and autoimmune disorders (Boehmer and Zanoni, 2025; Ivashkiv, 2018; Siebeler et al., 2023).

**Fig. 2:**
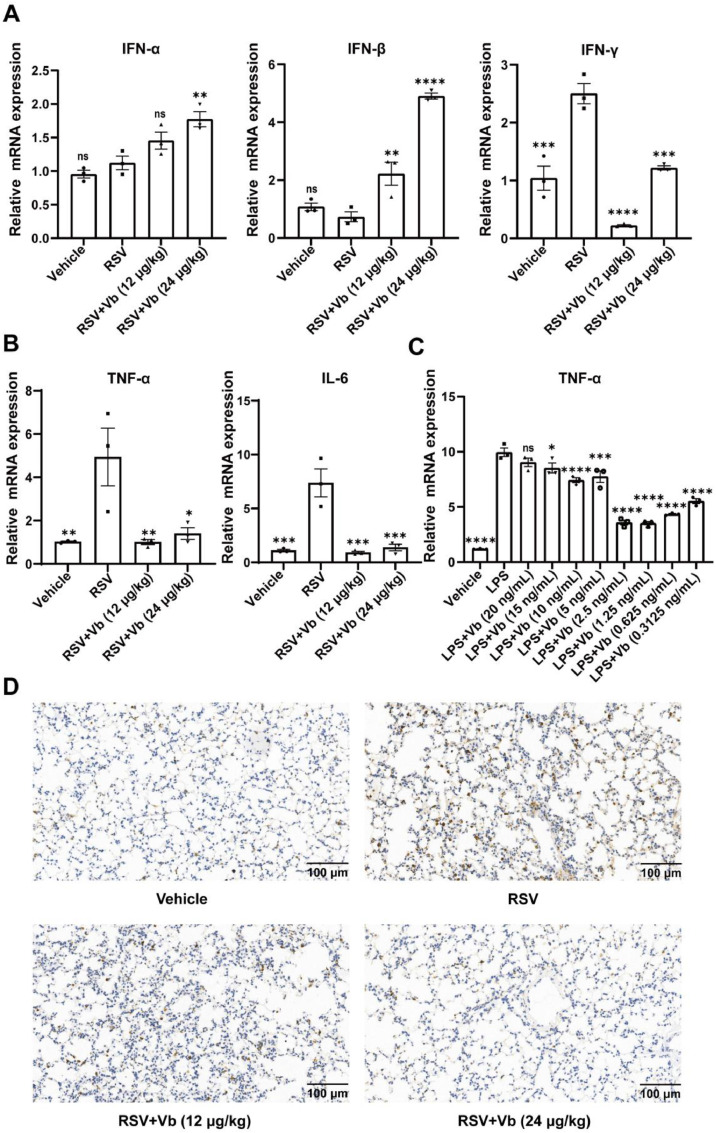
Effects of Vb on pro-inflammatory cytokine levels in lung tissues of RSV-infected mice. (A) mRNA expression levels of IFN-α, IFN-β and IFN-γ in murine lung tissues (*n* = 3). *P* < 0.05 indicates statistical significance versus the RSV model group, “ns” denotes no significant difference. (B) mRNA expression levels of TNF-α and IL-6 in murine lung tissues (*n* = 3). *P* < 0.05 indicates statistical significance versus the RSV model group. (C) Vb suppresses LPS-induced TNF-α mRNA expression in RAW 264.7 cells (*n* = 3). *P* < 0.05 indicates statistical significance versus the LPS group, “ns” denotes no significant difference. (D) Immunohistochemical staining of TNF-α in lung tissues. Scale bar: 100 μm. *P < 0.05, ^⁎⁎^*P* < 0.01, ^⁎⁎⁎^*P* < 0.001, ^⁎⁎⁎⁎^*P* < 0.0001.

In this study, we experimentally verified whether Vb influences the production of interferons in RSV-infected mice. The results revealed that Vb treatment increased the mRNA expression of type I interferons (IFN-α and IFN-β) while reducing the type II interferon (IFN-γ) in the lung tissues of RSV-infected mice (Fig. 2A). This distinct modulation suggests that Vb not only bolstering the direct antiviral defense mediated by type I IFNs but also attenuating excessive inflammation by suppressing type II IFN signaling.

Simultaneously, we also observed that, consistent with the histopathological findings, quantitative analysis of pro-inflammatory gene expression in murine lung tissues revealed that Vb treatment significantly downregulated mRNA levels of the inflammatory cytokines TNF-α and IL-6 in RSV-infected mice (Fig. 2B). Furthermore, we examined its effect on inflammatory cytokine production in LPS-stimulated RAW 264.7 macrophages. In this model, Vb significantly reduced LPS induced TNF-α mRNA level without significant cytotoxicity (Fig. 2C, Supplementary Fig. S1 B). This direct anti-inflammatory effect is consistent with our in vivo observations. Immunohistochemical analysis further confirmed that Vb downregulated TNF-α expression in lung tissues of RSV-infected mice (Fig. 2D).

**Fig. 3:**
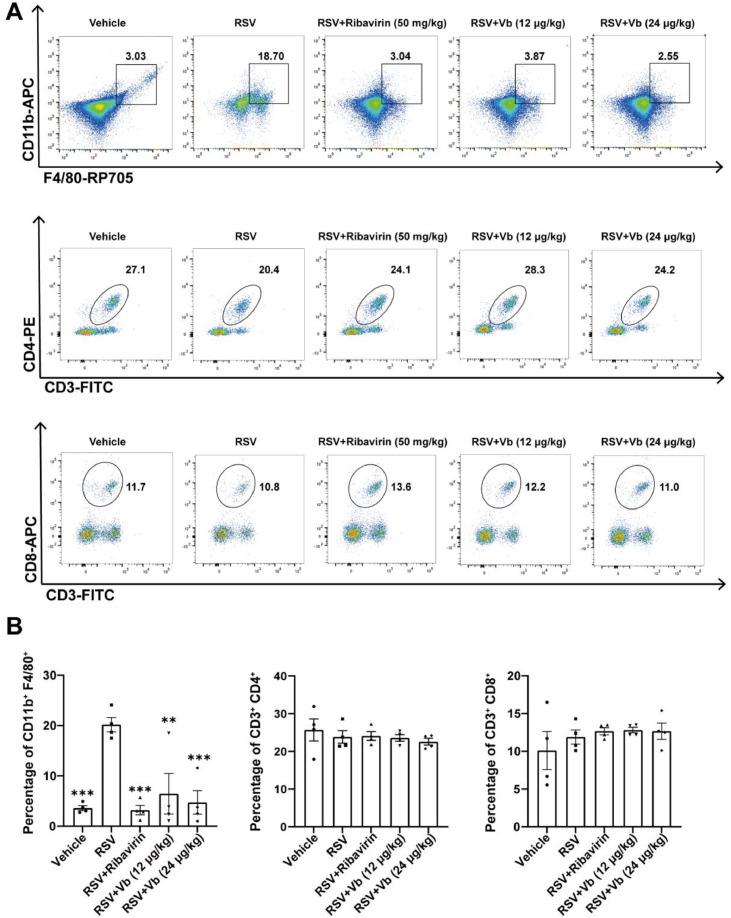
Flow cytometry analysis of inflammatory cells in the lung tissues of RSV-infected mice. (A) Representative flow cytometry plots showing macrophages, CD4^+^*T* cells and CD8^+^*T* cells in the lung tissues. (B) Quantification of the percentages of macrophages, CD4^+^*T* cells and CD8^+^*T* cells in the lung tissues (*n* = 4). *P* < 0.05 indicates statistical significance versus the RSV model group; ^⁎⁎^*P* < 0.05, ^⁎⁎⁎^*P* < 0.001.

During the early stages of viral pneumonia, macrophage infiltration is a critical component of the innate immune response required for viral clearance. However, excessive or sustained macrophage activation can lead to hyperinflammation and contribute to immunopathological lung damage. To evaluate the immunomodulatory effects of Vb in RSV-infected lungs, we performed flow cytometric analysis of pulmonary immune cell populations. Results showed that Vb treatment at doses of 12 μg/kg and 24 μg/kg significantly reduced macrophage infiltration into lung tissues of RSV-infected mice, while exerting no significant effects on CD4^+^ or CD8^+^ *T* cell recruitment (Fig. 3A,B).

Collectively, these results suggest that Vb ameliorates RSV-induced viral pneumonia, at least in part, by enhancing type I IFN-mediated antiviral defense, suppressing excessive pro-inflammatory responses and limiting macrophage-driven immunopathology.

### 3.3 Vb inhibits the NLRP3 inflammasome pathway in RSV-induced viral pneumonia in mice

**Fig. 4:**
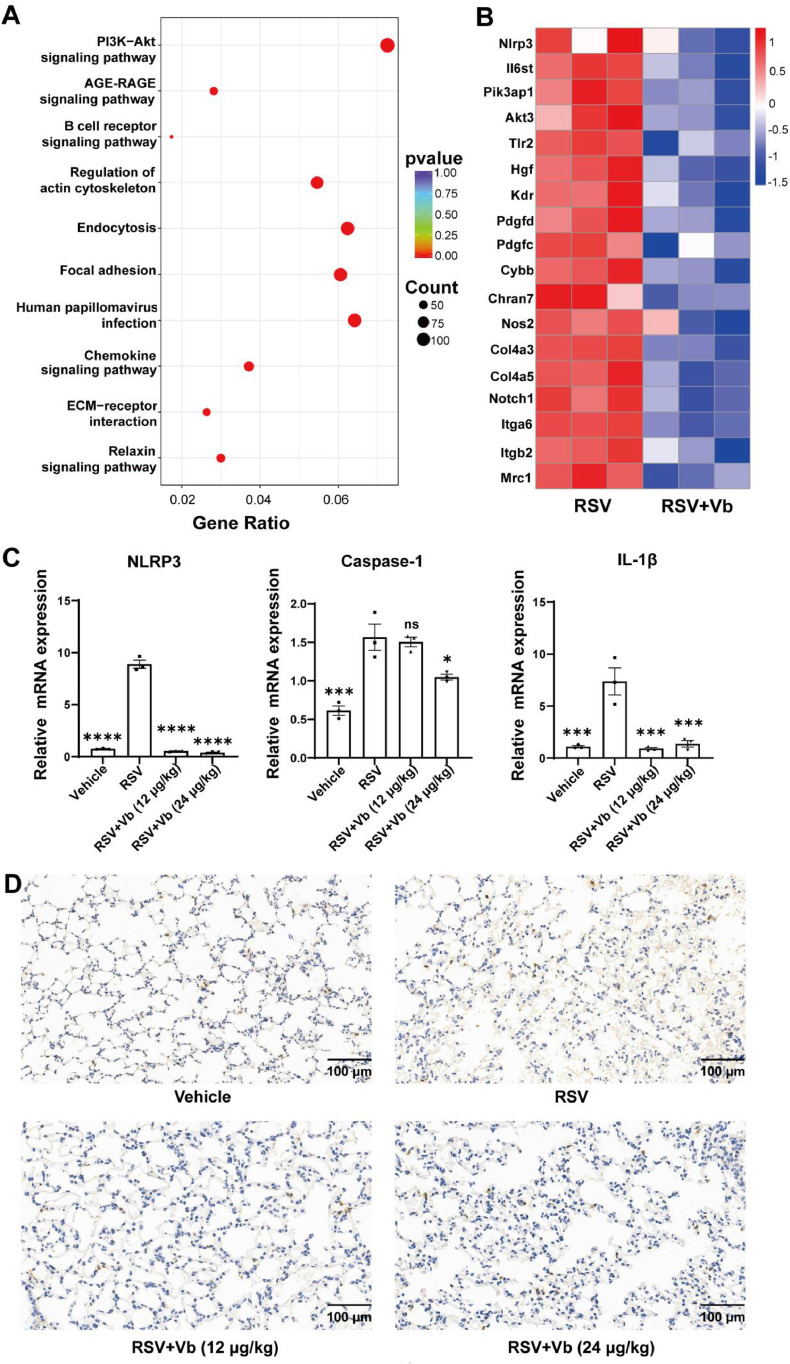
Effects of Vb on NLRP3 inflammasome-related gene expression in lung tissues of RSV-infected mice. (A) KEGG pathway enrichment analysis of downregulated genes in the Vb (24 μg/kg) treatment group relative to the RSV model group. RSV group vs Vb treatment group, *n* = 3. (B) Heatmap of key genes significantly downregulated by Vb treatment compared to the RSV model group. RSV group vs Vb treatment group, *n* = 3, *P* < 0.05. (C) mRNA expression levels of NLRP3, Caspase-1 and IL-1β in lung tissues (*n* = 3). *P* < 0.05 indicates statistical significance versus the RSV model group, “ns” denotes no significant difference, * *P* < 0.05, ^⁎⁎⁎^*P* < 0.001, ^⁎⁎⁎⁎^*P* < 0.0001. (D) Immunohistochemical staining of IL-1β in lung tissues. Scale bar: 100 μm.

To further elucidate the protective mechanism of Vb in the lung tissue of RSV-infected mice, we performed RNA sequencing. The results revealed that Vb treatment significantly downregulated genes enriched in inflammation, immunity, and cell survival-related pathways. KEGG enrichment indicated notable suppression of the PI3K-Akt pathway, along with AGE-RAGE, B cell receptor, and endocytosis signaling (Fig. 4A). Consistent with this, key inflammatory and immune genes, such as Nlrp3, Il6st, Tlr2, Pik3ap1, and Akt3, were markedly reduced in the Vb-treated group compared with the RSV model group (Fig. 4B).

RSV infection is known to induce excessive activation of the NLRP3 inflammasome, leading to inflammatory lung injury. We then assessed the expression levels of NLRP3 inflammasome pathway-related genes using RT-qPCR. The results revealed that Vb treatment significantly suppressed mRNA levels of NLRP3, Caspase-1, and IL-1β in lung tissues of RSV-infected mice with pneumonia (Fig. 4C). Immunohistochemical analysis further confirmed that Vb downregulated IL-1β expression in the lung tissues of RSV-infected mice (Fig. 4D). These findings were highly consistent with the RNA sequencing data, suggesting that Vb mitigates RSV-induced pulmonary inflammation, at least in part, by suppressing NLRP3 inflammasome activation, thereby attenuating downstream inflammatory cascades.

### 3.4 Bufalin, cinobufagin and resibufogenin are likely the primary active constituents responsible for the therapeutic effects of Vb against RSV-induced pneumonia

**Fig. 5:**
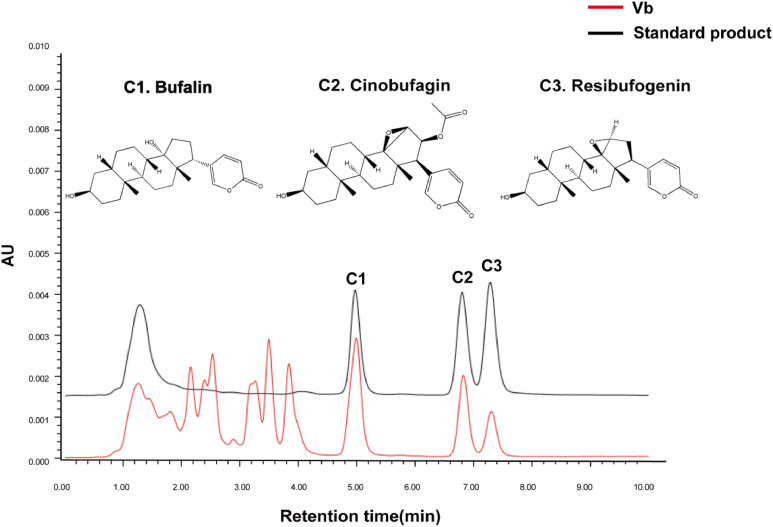
The major constituents of Vb used in this study were identified by HPLC.

To identify the main constituents of Vb, we performed chemical characterization using HPLC method. The chromatographic analysis revealed that bufalin (C_24_H_34_O_4_), cinobufagin (C_26_H_34_O_6_) and resibufogenin (C_24_H_32_O_4_) are the main constituents of Vb used in this study (Fig. 5).

**Fig. 6:**
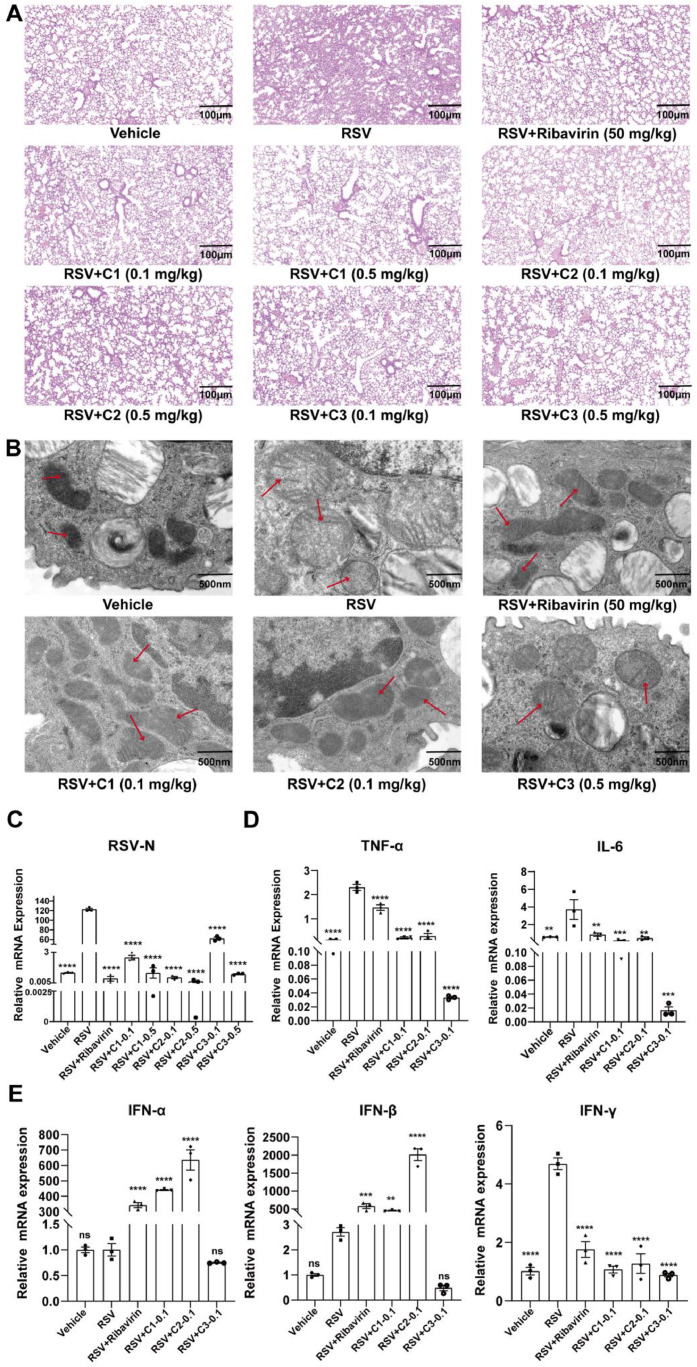
The major constituents of VB (bufalin, cinobufagin, and resibufogenin) alleviate RSV-induced viral pneumonia in mice. (A) Representative H&E-stained lung tissue sections. Scale bar: 100 μm. (B) Representative TEM images of lung tissues. Scale bar: 500 nm. The red arrows point to the mitochondria in the cytoplasm of type II pneumocytes. (C) mRNA expression level of RSV-N protein in murine lung tissue (*n* = 3). (D) mRNA expression levels of TNF-α and IL-6 in murine lung tissue (*n* = 3). (E) mRNA expression levels of IFN-γ, IFN-α and IFN-β in murine lung tissues (*n* = 3). C1: Bufalin, C2: Cinobufagin, C3: Resibufogenin; 0.1: 0.1 mg/kg, 0.5: 0.5 mg/kg. *P* < 0.05 indicates statistical significance versus the RSV model group; ^⁎⁎^*P* < 0.05, ^⁎⁎⁎^*P* < 0.001, ^⁎⁎⁎⁎^*P* < 0.0001.

Using the same RSV pneumonia model and treatment protocols as in prior experiments, we evaluated the therapeutic effects of these main constituents against RSV-induced viral pneumonia in vivo. H&E staining demonstrated that treatment with bufalin, cinobufagin or resibufogenin (all at doses of 0.1 mg/kg and 0.5 mg/kg) significantly reduced inflammatory cell infiltration in the lung tissues of RSV-infected C57BL/6 mice, ameliorating alveolar capillary congestion, edema, and overall pulmonary injury (Fig. 6A). TEM analysis further confirmed that all three constituents significantly ameliorated RSV-induced mitochondrial damage in alveolar epithelial cells (Fig. 6B).

**Fig. 7:**
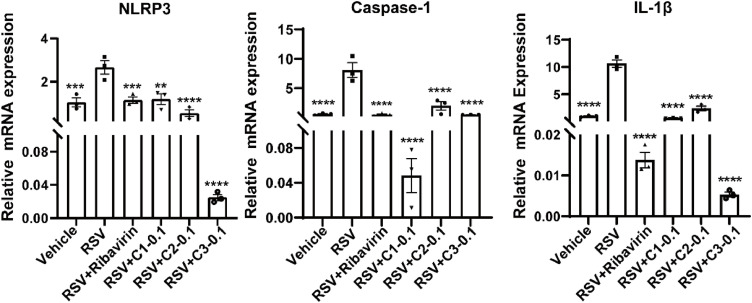
The effect of bufalin, cinobufagin, resibufogenin on the mRNA expression levels of NLRP3, Caspase-1 and IL-1β in the lung tissue of RSV infected mice. C1: Bufalin, C2: Cinobufagin, C3: Resibufogenin (*n* = 3); 0.1: 0.1 mg/kg. *P* < 0.05 indicates statistical significance versus the RSV model group; ^⁎⁎^*P* < 0.05, ^⁎⁎⁎^*P* < 0.001, ^⁎⁎⁎⁎^*P* < 0.0001.

To assess their antiviral and anti-inflammatory activities in vivo, we measured mRNA expression levels of viral genes and cytokines in the lung tissues by RT-qPCR. The results demonstrated that treatment with 0.1 mg/kg or 0.5 mg/kg bufalin, cinobufagin and resibufogenin significantly reduced mRNA expression level of RSV-N protein (Fig. 6C). At a low dose of 0.1 mg/kg, all three active components (bufalin, cinobufagin and resibufogenin) suppressed the mRNA expression of pro-inflammatory cytokines (TNF-α and IL-6) in the lungs of RSV-infected mice (Fig. 6D). These components also modulated the interferon response. All three active components reduced the mRNA expression of type II interferon (IFN-γ) mRNA, whereas bufalin and cinobufagin enhanced type I interferons (IFN-α and IFN-β) expression (Fig. 6E). Moreover, quantification of NLRP3 inflammasome pathway-related gene expression revealed that bufalin, cinobufagin and resibufogenin significantly down-regulated the mRNA levels of NLRP3, Caspase-1, and IL-1β (Fig. 7).

**Fig. 8:**
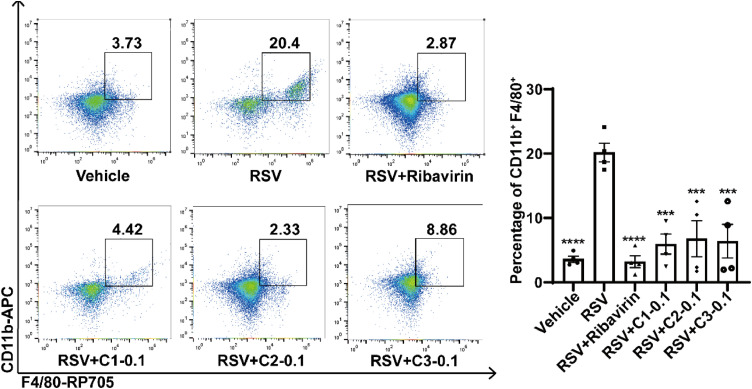
Flow cytometry analysis of macrophages in the lung tissues from RSV infected mice treated with vehicle control or major constituents of Vb. (A) The representative flow cytometry plots showing macrophages. (B) Percentages of macrophages in the lung tissues (*n* = 4). C1: Bufalin, C2: Cinobufagin, C3: Resibufogenin; 0.1: 0.1 mg/kg. *P* < 0.05 indicates statistical significance compared to the RSV model group; ^⁎⁎⁎^*P* < 0.001, ^⁎⁎⁎⁎^*P* < 0.0001.

In parallel, we employed flow cytometry to evaluate the regulatory effects of bufalin, cinobufagin and resibufogenin on macrophage infiltration. The results demonstrated that all three constituents of Vb significantly reduced macrophage infiltration in the lung tissues of RSV-infected mice (Fig. 8).

Collectively, these results demonstrate that bufalin, cinobufagin and resibufogenin are likely the key active constituents in Vb responsible for its therapeutic effects against RSV-induced viral pneumonia by suppressing pro-inflammatory responses.

## 4 Discussion

RSV is a leading cause of lower respiratory tract infections and bronchiolitis in children and elderly populations worldwide (Matias et al., 2014; Meissner et al., 2004). Based on current research findings, the high prevalence of RSV infection is primarily observed in infant and young child populations, accounting for approximately 52.44 % of cases, followed by elderly populations at around 20.73 % (Ursin and Klein, 2021). Furthermore, epidemiological studies indicate that male children are more susceptible to severe outcomes from RSV compared to female (Gonik, 2019; Klein and Flanagan, 2016; Malinczak et al., 2019; Muenchhoff and Goulder, 2014; Ursin and Klein, 2021).

Currently, no specific therapeutic drugs are available for RSV. RSV infection frequently triggers severe pulmonary inflammation and excessive immune cell activation, leading to subsequent lung tissue damage and airway obstruction (Lee et al., 2017; Shen et al., 2019; Vázquez et al., 2019). In this study, RSV-infected mice exhibited marked inflammatory cell infiltration and enhanced viral replication in lung tissues, consistent with the typical features observed in established animal models of RSV infection.

Vb and its active constitute bufalin have been demonstrated to exhibit broad-spectrum antiviral activity (Burkard et al., 2015; Garcia-Dorival et al., 2014), but their effects against RSV remain unclear. In China, Vb has been clinically approved for treating acute and chronic pyogenic infections. However, there is insufficient scientific evidence regarding its efficacy against RSV-induced viral pneumonia. Therefore, we investigated the therapeutic effects of Vb and its primary constituents in RSV-infected mice with viral pneumonia.

The Vb preparation (Chansu injection) is an approved drug with an established clinical safety profile. Our previous preliminary clinical study involving severe COVID-19 patients (*n* = 25 treatment; *n* = 25 control) demonstrated that no obvious adverse events (e.g., systemic or local rash, gastrointestinal discomfort, abdominal pain, diarrhea, or new arrhythmia) occurred during Vb treatment (Hu et al., 2021). Considering the established safety profile of Vb preparation demonstrated in human studies, we did not preliminary toxicity or pharmacokinetic studies performed prior in the animal model.

Our study revealed that Vb significantly ameliorated histopathological lung injury in RSV-infected mice, reduced inflammatory cell infiltration, alleviated alveolar capillary congestion and edema, suppressed viral loads and decreased levels of pro-inflammatory cytokines (such as TNF-α, IL-6, IL-1β, IFN-γ). At the same time, the expression of the key antiviral mediator type I interferon (IFN-α/β) was increased. Vb effectively transformed the RSV infection environment from a state of high inflammation and damage to a state of effective virus clearance and tissue protection. However, in vitro experiments demonstrated no direct cytoprotective effect of Vb on RSV-infected Hep-2 cells, suggesting that its anti-RSV activity likely involves immunomodulatory mechanisms rather than direct antiviral action.

Mechanistic investigations revealed that Vb treatment led to a significant reduction in pulmonary macrophage infiltration and suppressed activation of the NLRP3 inflammasome pathway. Furthermore, HPLC identified three primary bioactive constituents—bufalin, cinobufagin, and resibufogenin—all of which exhibited comparable therapeutic efficacy in alleviating RSV-induced viral pneumonia in vivo. These findings suggest that these constituents likely constitute the primary pharmacological basis for the therapeutic effects of Vb.

Flow cytometric analysis further demonstrated that both Vb and its primary constituents significantly reduced the infiltration of macrophages in the lung tissues of RSV-infected mice. Macrophages, as pivotal innate immune responders, play a dual role during RSV infection. While essential for viral clearance, their persistent activation or prolonged presence (especially the pro-inflammatory M1 phenotype) can exacerbate pulmonary damage through excessive pro-inflammatory cytokine secretion. In this study, mice treated with Vb exhibited significantly reduced pulmonary macrophage infiltration. Moreover, in an LPS-induced RAW 264.7 macrophage inflammation model, Vb significantly suppressed TNF-α mRNA expression. These results collectively suggest that its mechanism may involve the regulation of macrophage activation and recruitment, thereby ameliorating pulmonary inflammation.

To further investigate the mechanism by which Vb alleviates RSV-induced lung injury, we conducted an in-depth exploration using RNA sequencing. The results not only validated that Vb treatment significantly reduced the mRNA expression of NLRP3 and IL-6 in the lungs of RSV-infected mice, which is consistent with our subsequent findings. Most importantly, KEGG pathway enrichment analysis pinpointed the PI3K-Akt signaling pathway as a pivotal upstream regulator. The PI3K-Akt pathway serves as a central signaling hub, which numerous studies have demonstrated can positively regulate NLRP3 inflammasome activation (Li et al., 2025; Mei et al., 2025; Wang et al., 2025; Zhao et al., 2025; Zhong et al., 2023). Consequently, we speculate that Vb attenuates the assembly and activation of the NLRP3 inflammasome by inhibiting PI3K-Akt activity.

The NLRP3 inflammasome has been identified as a pivotal inflammatory regulator during multiple viral infections, including RSV. Previous studies have demonstrated that RSV activates the host NLRP3 inflammasome pathway, triggering the release of pro-inflammatory cytokines such as IL-1β, which exacerbates pulmonary inflammatory responses (Theofani et al., 2019). Moreover, NLRP3 inflammasome activation demonstrates significant correlation with macrophage polarization (Shao et al., 2023). Upon activation, the NLRP3 inflammasome promotes caspase-1-dependent maturation and release of IL-1β and IL-18, which are canonical markers of M1-type macrophages that are intimately associated with pro-inflammatory response (Coll et al., 2015; Kim et al., 2025; Sharma and Kanneganti, 2021). Our results demonstrate that Vb and its constituents significantly downregulated the expression of NLRP3-related genes in RSV-infected lung tissues, suggesting their therapeutic effects may involve suppression of NLRP3 inflammasome activation to mitigate RSV-induced pulmonary inflammatory injury.

While our study did not include phenotypic characterization of macrophage polarization (e.g., M1 vs. M2) using specific surface markers such as CD86 or CD206, the observed reduction in total macrophage infiltration remains a critical finding. Given that NLRP3 inflammasome activation is closely associated with the pro-inflammatory characteristic of the macrophages (Shao et al., 2023; Sharma and Kanneganti, 2021), it is plausible that Vb and its primary constituents may exert their immunoregulatory effects through modulation of NLRP3 inflammasome activation in macrophages. However, this hypothesis warrants further validation using M1/M2-specific profiling in future studies.

Notably, Vb inhibited RSV-N mRNA expression in the lungs of infected mice in a dose-dependent manner. Furthermore, the high dose demonstrated superior efficacy in suppressing Caspase-1 and inducing type I interferons (IFN-α/β). In contrast, the suppression of numerous inflammatory factors, such as IL-6 and TNF-α, was not strictly dose-dependent. We infer that the low dose of Vb exerts its effect through broad immunomodulation to suppress inflammation, whereas the high dose enables a more potent and targeted augmentation of core anti-inflammatory/anti-viral pathways (e.g., inflammasome suppression) and key mechanisms (e.g., enhancement of the type I interferon response).

Collectively, Vb and its bioactive constituents bufalin, cinobufagin, and resibufogenin effectively ameliorate RSV-induced viral pneumonia in mice. This therapeutic effect is likely mediated through coordinated mechanisms, including the enhancement of the type I interferon (IFN-α/β) response, a reduction in pro-inflammatory macrophage infiltration, suppression of inflammatory cytokine expression, and inhibition of NLRP3 inflammasome activation in the lungs. Together, these results provide both theoretical and experimental evidence supporting the potential of Vb as a therapeutic agent for RSV-induced viral pneumonia.

Currently, other immunomodulatory agents, such as NLRP3 inhibitors (e.g., MCC950) (Coll et al., 2019; Malinczak et al., 2021) and STING agonists (e.g., Vadimezan and ADU-S100) (Decout et al., 2021; Liu et al., 2021; Zhang et al., 2025a), exert anti-inflammatory and antiviral effects through an immunomodulatory mechanism analogous to Vb, rather than through direct antiviral activity. However, they are also associated with adverse effects, including excessive inflammatory responses. For instance, STING agonists may provoke severe lung inflammation and other complications while exerting their antiviral activity (Zhang et al., 2025b).

Published studies consistently highlight the limited arsenal of effective therapeutics against respiratory syncytial virus (RSV). Current approaches are dominated by antibodies and vaccines directed against the RSV fusion (F) glycoprotein (McLellan, 2015; Oraby et al., 2025). Nevertheless, despite the overall conservation of RSV-F, sequence variations in specific epitopes are associated with antibody resistance (Jones et al., 2019). Consequently, such therapeutics have high risks of inducing viral resistance (Tang et al., 2019). In contrast to these direct-targeting strategies, our findings demonstrate that Vb operates by mobilizing the host's immune system, this approach confronts the virus with a broad, pleiotropic immune response. Therefore, we propose that Vb represents a promising therapeutic candidate, potentially effective against RSV strains that have developed resistance to conventional antiviral agents.

While this study confirms the efficacy of Vb in alleviating RSV-induced pneumonia through immunomodulation, the precise molecular mechanisms underlying these protective effects remain to be fully elucidated. Further investigation into the specific bioactive compounds of Vb and their molecular targets.

## Funding

This work was financially supported by the National Key Research and Development Program of China (2023YFC2308200), the Jiangsu Province Science and Technology Development Project of Traditional Chinese Medicine (No. ZT202110), the “New High School 20 Items” Project of Jinan (202333006), and the Taishan Scholars Program (tstp20231239).

## CRediT authorship contribution statement

**Hui Cai:** Writing – review & editing, Writing – original draft, Validation, Data curation. **Lijing Hou:** Writing – review & editing, Methodology, Data curation. **Jingjie Chang:** Validation, Data curation. **Qing Yang:** Validation, Data curation. **Mingming Wang:** Validation, Data curation. **Lin Wang:** Validation. **Yao Li:** Validation, Data curation. **Xueting Cai:** Supervision, Methodology. **Jie Yang:** Writing – review & editing, Supervision, Methodology, Conceptualization. **Peng Cao:** Writing – review & editing, Supervision, Methodology, Funding acquisition, Conceptualization. **Jiao Chen:** Writing – review & editing, Writing – original draft, Supervision, Methodology, Funding acquisition, Conceptualization.

## Declaration of competing interest

The authors declare that they have no known competing financial interests or personal relationships that could have appeared to influence the work reported in this paper.

## Data Availability

Data will be made available on request.

## Appendix Supplementary materials

Image, application 1

Image, application 2
