# Supplementary material for: Venenum bufonis and its active constituents alleviate RSV-induced pneumonia in mice by suppressing macrophage infiltration and NLRP3 inflammasome activation
Source: Virus Res. 2025 Dec 3;363:199672. doi: 10.1016/j.virusres.2025.199672 (PMC12743554; doi:10.1016/j.virusres.2025.199672)
Supplement: Supplementary file 2 [file mmc2.docx]

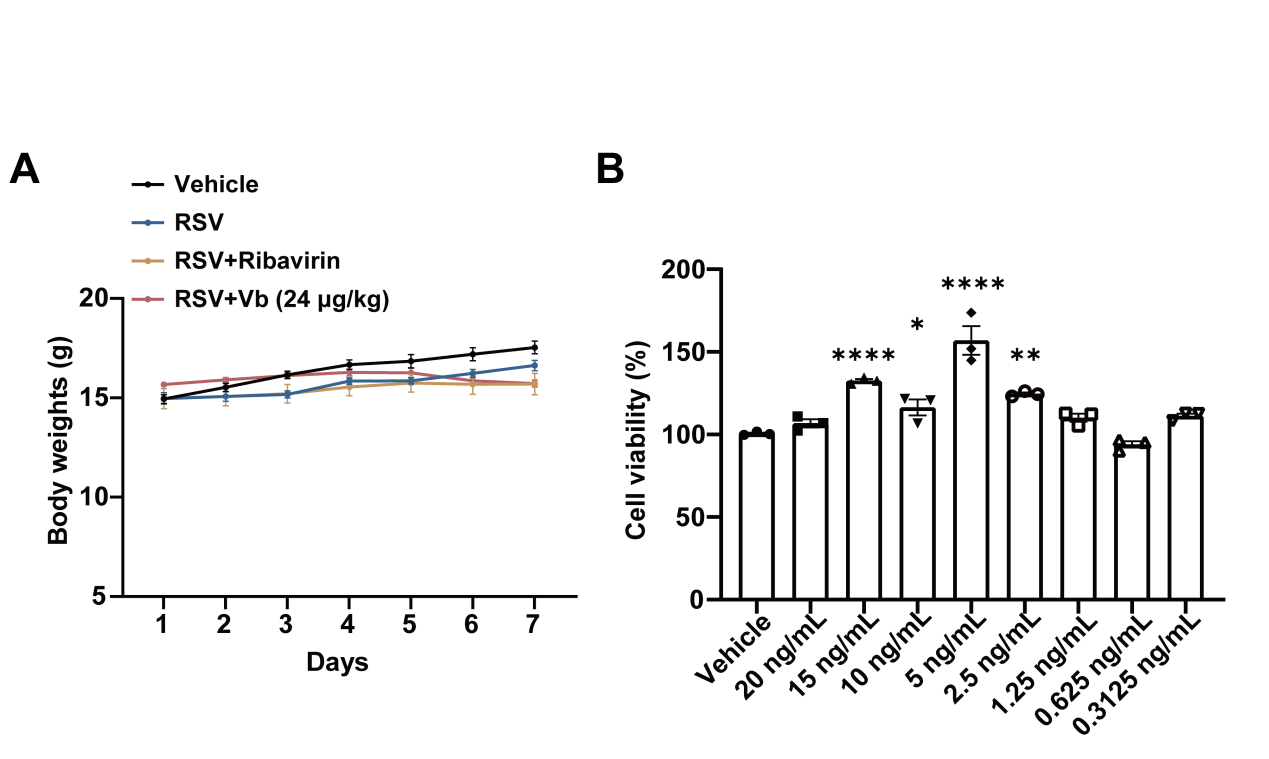


**Figure S1.** **Effects of Vb on body weight in mice and viability of RAW 264.7 cells.** (A) Body weight of C57BL/6 mice during the experimental period. (n=6/group). (B) Viability of RAW 264.7 cells following treatment with a gradient of Vb concentrations (20, 15, 10, 5, 2.5, 1.25, 0.625, 0.3125 ng/mL) for 24 hours, as measured by CCK-8 assay (n=3). *P* < 0.05 indicates statistical significance versus the Vehicle group. ^*^ *P* < 0.05, ^**^ *P* < 0.005, ^****^ *P* < 0.0001.
